# Structured Training in Robotic Abdominal Wall Surgery: A Systematic Review of Educational Models, Methodologies, Existing Gaps and Unmet Needs

**DOI:** 10.3389/jaws.2025.15190

**Published:** 2025-08-18

**Authors:** Francesco Brucchi, Annabelle De Troyer, Alice Gori, Gianlorenzo Dionigi, Eva Vanderstraeten, Alexander Mottrie, Isabelle Van Herzeele, Niki Rashidian, Filip Muysoms

**Affiliations:** ^1^ Department of General Surgery, University of Milan, Milan, Italy; ^2^ ORSI Academy, Melle, Belgium; ^3^ Department of General Surgery, AZ Maria Middelares, Ghent, Belgium; ^4^ Department of General Surgery, Ziekenhuis Aan de Stroom (ZAS) Antwerp, Antwerp, Belgium; ^5^ Department of General Surgery, University of Bologna, Bologna, Italy; ^6^ Division of General and Endocrine Surgery, Istituto Auxologico Italiano, IRCCS (Istituto di Ricovero e Cura a Carattere Scientifco), Milan, Italy; ^7^ Department of Pathophysiology and Transplantation, University of Milan, Milan, Italy; ^8^ Department of Thoracovascular Surgery, Ghent University Hospital, Ghent, Belgium; ^9^ Department of General, HPB Surgery, and Liver Transplantation, Ghent University Hospital, Ghent, Belgium

**Keywords:** robotic-assisted surgery, abdominal wall surgery, proficiency-based training, surgical training, structured curricula

## Abstract

**Background:**

Robotic-assisted abdominal wall surgery demands advanced technical proficiency. The advent of robotic platforms has driven the development of various training approaches, including simulation-based modules, animal models, and structured curricula. This systematic review critically assesses current training strategies and models, comparing their effectiveness in skill acquisition through validated assessment tools and evaluating their implementation from a cost-effectiveness perspective.

**Methods:**

A comprehensive search of the scientific literature was conducted across three major databases (PubMed, Embase, Cochrane, Google Scholar) up to April 2025. The study was registered in PROSPERO (CRD420251027155) and conducted in accordance with the Preferred Reporting Items for Systematic Reviews and Meta-Analyses (PRISMA) guidelines. Studies were selected based on inclusion of robotic training programs related to abdominal wall surgery.

**Results:**

Out of 3,038 records identified, 8 studies were included. The overall methodological quality was acceptable, with all studies showing moderate risk of bias. Training models varied and included virtual reality simulation (n = 4), inanimate models (n = 3), porcine models (n = 2), and intraoperative training (n = 4). Three studies described integrated, proficiency-based curricula. Skill acquisition was reported using validated tools such as GEARS, OSATS, or the Zwisch scale in only two studies. Reported costs ranged from €40 for silicone models to €600 for porcine models; one study demonstrated $1,207 in cost savings per case post-training.

**Conclusion:**

Current training models for robotic-assisted abdominal wall surgery are heterogeneous in design, assessment methods, and cost. While integrated curricula show promise, few studies employ validated tools to evaluate skill acquisition. Further high-quality research is needed to standardize training approaches and assess their cost-effectiveness.

## Introduction

Hernia repair, one of the most commonly performed surgeries worldwide in men and women, has become increasingly complex due to new techniques, more challenging cases, a recognized tailored approach, and growing public awareness that demands nothing less than optimal treatment results [1, 2]. The rapid expansion of robotic surgery in abdominal wall reconstruction has brought significant technical and anatomical advantages, but also increased procedural complexity [3]. Mastery now requires not only advanced minimally invasive skills and anatomical expertise, but also substantial robotic operative experience to achieve proficiency [4–6].

However, clinical exposure alone is often insufficient for young surgeons to achieve full autonomy within a reasonable timeframe, especially given the limited access to robotic platforms during training. The increasing emphasis on patient safety and procedural precision has highlighted the limitations of the traditional apprenticeship model, particularly in the context of robotic surgery [7]. A key challenge in modern surgical education is ensuring that training programs equip surgeons with the necessary skills to competently and safely integrate new robotic technologies into clinical practice [8, 9].

Proficiency-based progression (PBP) training offers a safe, structured alternative by requiring trainees to demonstrate competency in a safe environment, through validated metrics in simulated settings—using various models, e.g., virtual reality models [10], inanimate models [11], animal or cadaveric models [12, 13] —before operating on patients [14–18]. It involves evaluating performance against predefined quantitative metrics (benchmarks). In this process, learners must achieve a score that reflects the performance of experienced surgeons before they are allowed to progress to clinical surgery [19]. Despite the increasing recognition of its value, the literature still lacks comprehensive and validated training pathways specifically designed for robotic abdominal wall procedures, as well as shared and standardized metrics applicable on a global scale, highlighting a critical area for further research and development.

This systematic review aims to examine and compare different robotic surgical training methods and models used in abdominal wall procedures, particularly focusing on simulation and skill acquisition metrics. By evaluating current evidence, this review provides insight into the most effective training models in terms of skill transfer and cost-effectiveness and offers guidance for future educational integration.

## Methods

A comprehensive online systematic search was conducted using PubMed, Embase, Google Scholar and Cochrane databases for eligible articles until April 8, 2025. A combination of keywords was used in the search: “education,” “simulation training,” “training,” “teaching,” “preceptorship,” “curriculum,” “robotic surgery,” “robotic surgical procedures,” “Abdominal Wall Surgery,” “Hernia,” and “Incisional Hernia,” “Hernia Repair.” The detailed search strategies have been provided in the Supplementary Material (Supplementary Figure S1). This systematic review was reported in accordance with the PRISMA (Preferred Reporting Items for Systematic Reviews and Meta-Analyses) 2020 Statement [20], and was pre-registered with PROSPERO (registration number: CRD420251027155). The AMSTAR (A Measurement Tool to Assess Systematic Reviews) checklist is included in the Supplementary Material [21]. The research encompassed original scientific manuscripts, comparative studies and case series. The inclusion criteria were: (1) focus on robotic abdominal wall surgery and (2) description of a training pathway or simulation model. Exclusion criteria included: case reports, review articles, articles in non-English languages, articles unrelated to the review topic and training programs not focusing on robotic abdominal wall surgery. Duplicates were excluded, including both articles replicated across multiple databases and studies analyzing the same cohort, to prevent data overlap.

The selection process was conducted blindly by two reviewers, who independently reviewed the titles and abstracts of each article and subsequently assessed the full-text articles against the predetermined eligibility criteria. Disagreements were resolved through discussion or, if unresolved, through arbitration with a third reviewer.

### Quality Assessment

(Methodological quality of the included studies was graded using the Medical Education Research Study Quality Instrument (MERSQI) [22]. Two investigators independently assessed the risk of bias for all studies. Disagreements were resolved through discussion or, if unresolved, through arbitration with a third reviewer. Relevant articles were also reviewed and summarized through the perspective of the Kirkpatrick’s Evaluation framework to determine the effectiveness, strength, and weaknesses of the training programs [23]. The Kirkpatrick’s model focuses on evaluating how trainees are reacting to the program, what they are learning from the program, how this is changing their behavior upon entry into practice, and finally the results the training programs are having on outcomes. A detailed overview of the MERSQI and Kirkpatrick assessment frameworks, including their domains and scoring systems, is provided in Supplementary Table S1.

## Results

### Study Selection Process

**FIGURE 1:**
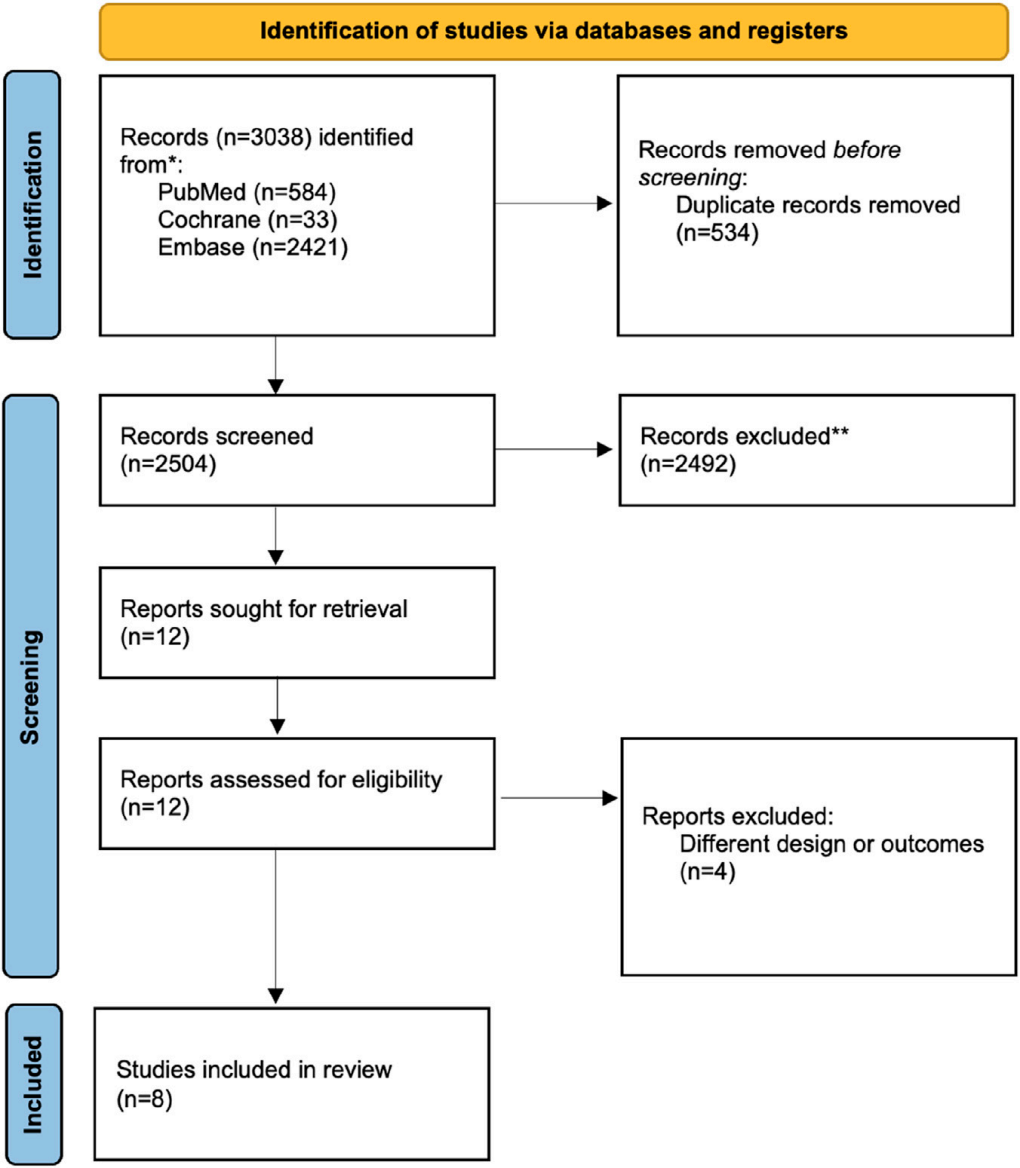
Flowchart of study screening according to PRISMA guidelines.

Figure 1 shows the flow of studies through the screening process. The literature search identified a total of 3,038 records from three major databases: PubMed (n = 584), Cochrane Library (n = 33), and Embase (n = 2,421). After removal of 534 duplicates, 2,504 records were screened by title and abstract. Following screening, 12 full-text articles were retrieved and assessed for eligibility. Of these, 4 studies [24–27] were excluded due to differing designs or lack of relevant outcomes, resulting in 8 studies being included in the final systematic review [12, 28–34].

### Study Quality and Risk of Bias

The Supplementary Table S1, summarizes the quality criteria assessed for each study using the MERSQI tool. The overall quality of the studies was acceptable, with all assessed as having a moderate risk of bias. The overall mean score of the studies was 10 (IQR = 10). Training effectiveness was categorized using Kirkpatrick’s model. Most studies [12, 28, 29, 32, 33] reached Level 1 or 2, focusing on fidelity and technical performance. Only Tam et al. [34] reported clinical outcomes (Level 4), while Mustafa et al. and Madureira et al. demonstrated behavioral changes (Level 3) [30, 31]. See Table 1 for details.

**TABLE 1:** Characteristics of selected studies.

Author	Country	Enrollment years	Study design	MERSQI scale	Kirkpatrick level
Hays et al. [29]	USA	2019–2021	Retrospective	12	2
Vierstraete et al. [12]	Belgium	NR	Descriptive Developmental Model	6	1
Gonçalves et al. [24]	Portugal	NR	Prospective	11	1
Ollapallil Jacob et al. [32]	Australia	NR	Descriptive Developmental Model	5	1
Ebeling et al. [33]	USA	2017–2019	Retrospective	9	2
Tam et al. [34]	USA	2015–2017	Retrospective	15	4
Mustafa et al. [30]	USA	2013–2017	Retrospective	9	3
Madureira et al. [31]	Brazil	2012–2015	Retrospective	11	3

### Characteristics of Included Studies

The included studies, published between 2017 and 2024, evaluated various robotic training strategies for abdominal wall surgery. Study designs, participant types, and key findings are summarized in Tables 1, 2. Most studies focused on rTAPP procedures [12, 28, 33, 34], while a few included ventral hernia repair [29–31] or more complex techniques such as eTEP or TAR [32]. Study populations ranged from residents to attending surgeons.

**TABLE 2:** Characteristics of the training models or pathways and key findings of the included studies.

Study	Training model	Training pathway	Population	Outcome metrics	Key findings
Hays et al. [29]	Simulation-based robotic curriculum (IHR & VHR drills) and video review	Inanimate drills with OSATS scoring	PGY-3 general surgery residents (n = 20)	OSATS scores, time to completion	Significant improvement in OSATS and operative time over 4 attempts; residents reached attending-level scores for VHR
Vierstraete et al. [12]	SPIRIT model (porcine rTAPP IHR)	Live animal training + structured protocol, defined procedural phases and steps	NR	Feasibility, anatomical fidelity	Structured steps mimicking human IHR; high fidelity and reproducibility for robotic IHR training
Gonçalves et al. [24]	RAWS4all project hands-on training (rTAPP)	Structured workshop (DaVinci simulator and silicone model)	25 residents and surgeons naive to robotic surgery	Fidelity questionnaire, score for execution, quality and global performance	1. Very high fidelity of the model 2. Execution, quality, and global performance was higher in the senior’s group
Ollapallil Jacob et al. [32]	Porcine model for advanced abdominal wall dissections (eTEP and TAR)	Live animal training	2 consultants	Feasibility, anatomical landmarks realism, time to completion	Porcine model provides high fidelity simulation for complex wall dissections
Ebeling et al. [33]	Resident training in rTAPP	Prospective observational study using intraoperative performance tools	27 residents and 2 consultants	Total autonomy and autonomy for each segment (GEARS, total GEARS and Zwisch scale)	Residents with >30 robotic console cases had significantly higher competency and autonomy scores. Skill gains were evident after 10 cases. Autonomy varied by procedure step. Competency did not equate to proficiency at low volumes
Tam et al. [34]	Proficiency-based credentialing (rTAPP)	Simulation + inanimate biotissue + live proctoring	16 surgeons	Operative time, hospital cost saving with program implementation	Trained surgeons using a structured proficiency-based robotics training curriculum had shorter operative times and $1200 less cost per case
Mustafa et al. (2018)	Integrated robotic curriculum: online module, bedside teaching, simulator tasks (≥90% score), 10 bedside assists, progressive console participation, and full case completion under supervision. (VHR and IHR)	Retrospective pre/post curriculum comparison	General surgery residents	Case distribution, robotic case volume	Robotic training increased MIS case exposure, particularly hernia cases
Madureira et al. (2017)	Multi-stage robotic surgery training (VHR and IHR)	Simulation + Live Surgery: (1) Online module on robot functions, (2) Mimic simulator for dexterity skills, (3) Dry-lab training on the robot with models, (4) Cadaver or live animal training at Intuitive Surgical centers before clinical cases	General surgeons (n = 8) and urologists (n = 5)	Case distribution, complication rates	Safe implementation with low complication rates across 293 robotic procedures, including abdominal wall

Study	Procedure divided in phases and/or steps	Benchmark	Curriculum perception	Transferability of skills in the OR	Cost/savings of the program/model
Hays et al. [29]	No	3 attendings’ median OSATS score	4 out of 5 in utility based on a Likert scale	No	NR
Vierstraete et al. [12]	Yes	No	NR	No	600 euros/model (excluding VAT)
Gonçalves et al. [24]	Yes	NR	Participants strongly agreed that the model is adequate	No	40 euros/model (single use)
Ollapallil Jacob et al. [32]	Yes	No	NR	No	NR
Ebeling et al. [33]	Yes	No	NR	Yes	NR
Tam et al. [34]	No	No	NR	Yes	$1,207 saved per robotic hernia case
Mustafa et al. (2018)	No	No	NR	Yes	NR
Madureira et al. (2017)	No	No	NR	Yes	NR

### Training Modalities

Training models varied across studies and generally fell into the following categories:

• Digital Simulation-Based/Virtual Reality Training (n = 4):

Gonçalves et al. [28] and Tam et al. [34] introduced virtual simulation as the initial step before hands-on practice. Mustafa et al [31]. and Madureira et al. [30] began with e-learning modules, followed by simulation-based dexterity training at the robotic console.

• Inanimate models (n = 3):

Hays et al. [29] used inanimate drills with OSATS scoring showing significant improvements in both time to task completion and technical skills. Gonçalves et al. [28] reported high-fidelity silicone models in a structured hands-on course. Tam et al. [34] employed a detailed 3D anatomical replica for simulation training.

• Animal Models (n = 2):

Vierstraete et al. [12] and Ollapallil Jacob et al. [32] utilized anesthetized porcine models to simulate robotic inguinal hernia repair. These models were highly rated by experts for anatomical fidelity and procedural realism. Notably, Vierstraete reported a cost of nearly €600 per anesthetized pig, and Ollapallil highlighted the porcine model as significantly less expensive than cadaveric alternatives.

• Intraoperative training (n = 4):

Four studies included intraoperative training [30, 31, 33, 34]. Ebeling et al. assessed autonomy across procedural segments, while Tam, Mustafa, and Madureira placed intraoperative exposure at the end of simulation-based pathways.

• Integrated/Proficiency-Based Curricula (n = 3):

Tam, Mustafa, and Madureira et al. [30, 31, 34] implemented comprehensive pathways combining simulation, inanimate models, and supervised console time. Only Tam and Mustafa included proficiency-based steps, with the latter study requiring ≥90% scores on simulator tasks before progression.

### Skill Acquisition and Assessment Tools

Among the included studies, only Hays et al. [29] and Ebeling et al. [33] reported formal performance assessments tools for trainees:

• GEARS (Global Evaluative Assessment of Robotic Skills) tool [35] was used by Ebeling et al. [33] to objectively assess technical performance across key domains such as depth perception, bimanual dexterity, efficiency, force sensitivity, autonomy, and robotic control. The scale was used both globally and within each of the four procedural segments.
• Zwisch scale [36]: a four-point Likert scale for grading resident autonomy. This scale was used alongside the GEARS tool by Ebeling et al. to strengthen the validity of their performance assessments, providing an additional measure of intraoperative autonomy.
• OSATS (Objective Structured Assessment of Technical Skill) [37] was employed by Hayes et al. [29] to evaluate technical proficiency using structured checklists and global rating scales across key surgical competencies such as: gentleness, time and motion, instrument handling, flow of operation, tissue exposure, and summary score. This study also included a benchmark OSATS score, established using assessments from three attending surgeons.

### Cost Considerations

Cost-related data were inconsistently reported across the included studies and are summarized in Table 2.

Ultimately, only Tam et al. [34] reported cost savings associated with the implementation of a robotic training program; however, the study did not specify the costs related to the development and structuring of the program itself. The remaining articles reported only the cost of training materials.

## Discussion

To our knowledge, this is the first systematic review to focus specifically on training strategies in robotic abdominal wall surgery, and it may serve to guide the development of structured curricula, standardization efforts, and future research in this evolving field. While the growing complexity of these procedures has generated increasing interest in structured and simulation-based training, the current literature remains limited and heterogeneous, with relatively few studies offering validated or standardized approaches. This review highlights the variety of educational models being explored and underscores the need for more robust, evidence-based curricula to support skill acquisition in this evolving field.

A key finding from this review is the limited number and heterogeneous nature of training approaches, with most programs integrating multiple components [30, 31, 34]—such as digital simulation, inanimate or animal models, and intraoperative experience. Notably, digital simulation and virtual reality training emerged as a foundational element in four studies [28, 30, 31, 34], typically serving as the initial step of the pathway. This approach supports early cognitive and psychomotor development in a safe environment, with several robotic platforms offering structured feedback and progression tracking.

Both inanimate and animal models offer unique advantages and limitations in robotic surgical training. Inanimate models, such as the silicone systems used by Gonçalves et al. and Tam et al. [28, 34], provide low-cost, highly reproducible platforms, supporting repeatable, tactile practice that can facilitate the transition to live surgery. However, they lack the ability to simulate critical surgical maneuvers like coagulation or handle the dynamic tissue interactions of a real operative field. As such, they may be more appropriate for the initial phases of the training pathway.

Animal models, particularly the porcine models described by Vierstraete et al. and Ollapallil Jacob et al. [12, 32], offer higher anatomical fidelity and procedural realism but a higher cost when compared to inanimate ones, making them better suited for advanced trainees approaching full clinical practice and after a first step training on virtual reality and/or inanimate models. Animal models are particularly valuable for complex dissection training, though their use is often limited by higher costs, ethical considerations, and logistical challenges. Together, these two approaches provide complementary, stage-appropriate training options, allowing for progressive skill acquisition as trainees move from basic technical tasks to more complex, high-stakes procedures.

Intraoperative training was integrated in four studies [30, 31, 33, 34], with varied implementation. Ebeling et al. [33] used intraoperative exposure as a structured, progressive assessment tool, while Tam et al. [34], Mustafa et al. [31] and Madureira et al. [30] placed it at the end of a graduated pathway, following simulation and lab-based preparation. This reflects a broader trend toward stepwise progression from low- to high-fidelity environments, supporting the concept of deliberate practice.

PBP training has emerged as a transformative approach in robotic surgical education, emphasizing the attainment of specific performance benchmarks before trainees advance to subsequent stages. In a multicenter randomized controlled trial, De Groote et al. demonstrated that PBP training significantly improved robotic suturing and knot-tying skills among surgical residents [38]. Participants in the PBP group were approximately ten times more likely to achieve predefined proficiency benchmarks compared to those undergoing conventional training, with a notable 51% reduction in performance errors [38]. In this systematic review, only three studies adopted an integrated curriculum, combining digital, inanimate, and operative experiences into a sequential training structure [30, 31, 34]. Among them, Mustafa et al. [31] was the only study to define proficiency-based benchmarks, requiring minimum simulator performance thresholds (≥90%) before advancing. Such models align with the principles of PBP training and may help standardize robotic abdominal wall surgery credentialing.

Finally, the success of any surgical training program relies not only on the quality of its models but also on the competence and consistency of its instructors. Faculty training and ongoing calibration are essential to ensure that learners receive standardized, high-quality guidance. However, this consistency is challenging to achieve, given the variability in hospital resources, faculty experience, and access to robotic platforms across institutions. This variability may lead to inconsistent educational outcomes and highlights the need for faculty development programs alongside training curricula [39].

Despite the increasing emphasis on simulation and structured curricula, only Hays et al. [29] and Ebeling et al. [33] reported formal assessments of trainee performance. Tools such as GEARS [35], Zwisch scale [36], and OSATS [37] provide validated, objective metrics for technical skill and autonomy. Ebeling et al. [33] notably applied these tools both globally and segmentally within the surgical procedure, offering a nuanced view of skill acquisition across specific phases. Hays et al. [29] also included benchmark OSATS scores from expert surgeons, reinforcing the potential for structured simulation to mimic real-world expectations. Gonçalves et al. [28] reported having assessed trainees based on a stepwise breakdown of the procedure and the identification of major and minor errors; however, the details of this assessment process were not clearly described.

This underlines a key gap in the literature: while many programs adopt training tools, fewer rigorously evaluate outcomes using validated metrics. The lack of widespread, standardized assessment may hinder the ability to compare or benchmark training effectiveness across institutions.

Cost reporting was inconsistent among studies, though several models demonstrated economic feasibility. For instance, Gonçalves et al. described a single-use silicone model costing €40, while Vierstraete et al. [12] reported €600 per porcine model. Importantly, Tam et al. [34] demonstrated measurable institutional savings, reporting $1,207 (−20.1%) saved per case following implementation of a structured robotic training program. Specifically, the procedural cost for participants who did not undergo the curriculum was $6,009.42, compared to $4,802.23 for those who completed the training. The most notable cost reductions were observed in support unit expenses ($939.82 vs. $585.35, −37.7%) and anesthesia costs ($1,208.25 vs. $852.16, −29.5%).

These findings support the idea that initial investments in training may be offset by increased efficiency, reduced operative times, and improved outcomes.

Most studies included in this review were conducted in high-resource settings across Europe, North America, and Australia, where access to robotic systems, simulation centers, and expert faculty is more readily available. This geographic concentration may limit the generalizability of the findings to low- and middle-income countries, where the cost and infrastructure requirements for robotic training may present significant barriers. As robotic surgery continues to expand globally, it will be crucial to develop training models that are adaptable to resource-limited contexts and supported by international collaboration.

Nonetheless, more robust cost-effectiveness analyses are needed to better inform institutional decisions regarding simulation infrastructure and program design.

The findings of this review underscore the educational value of structured training in robotic abdominal wall surgery, particularly in enhancing operative performance and progressing toward autonomy. Several studies demonstrated that skill acquisition can begin early in the training process, but consistent, high-level performance typically requires greater case volume. Notably, autonomy appears to develop in a stepwise manner, with specific procedural segments demanding distinct thresholds of competency—highlighting the need for task-specific evaluation within training curricula.

**FIGURE 2:**
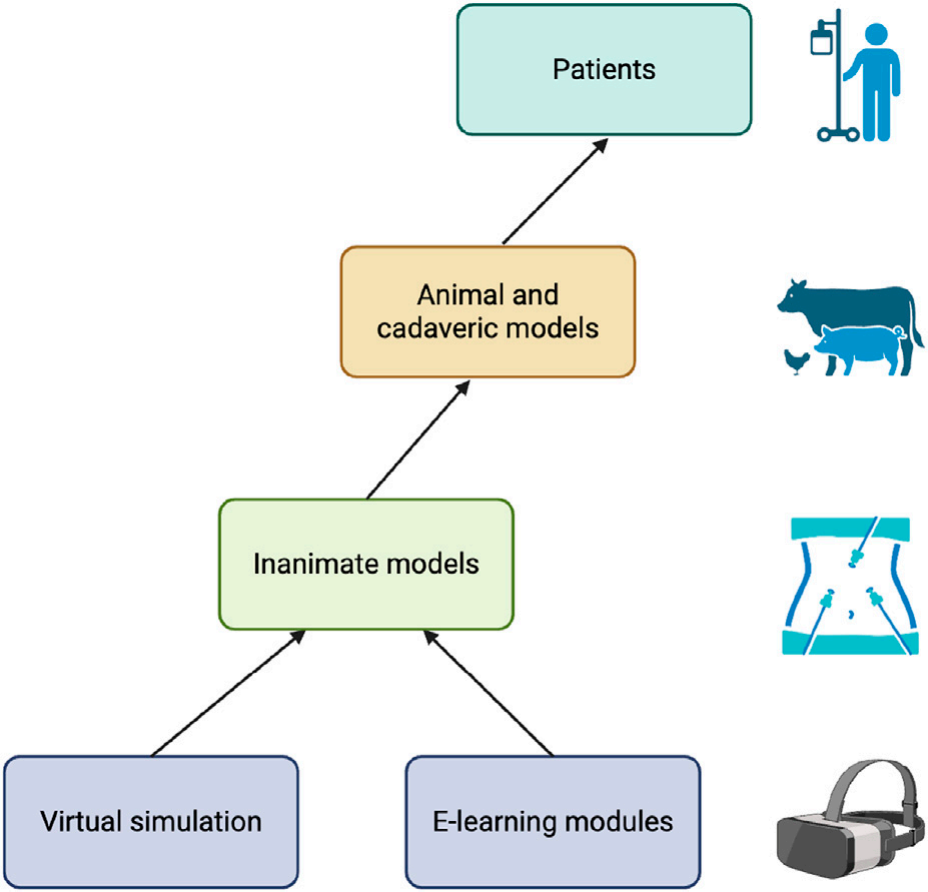
Proposal for a progressive, multimodal training pathway.

Based on these insights, we propose a progressive, multimodal training pathway that begins with foundational cognitive and psychomotor skills acquired through e-learning modules and virtual simulation (Figure 2). These initial stages are followed by inanimate model practice, which supports the consolidation of basic technical tasks. More advanced and realistic training is then pursued using animal and cadaveric models, culminating in supervised clinical practice on patients. This structured progression aligns with a proficiency-based model and is designed to both shorten the learning curve and ensure safe, autonomous surgical performance.

Importantly, such a stepwise framework also allows for the rational allocation of resources. Given the significantly higher costs and logistical constraints associated with cadaveric and animal models, their use should be reserved for trainees who have already achieved a sufficient level of proficiency in lower-fidelity settings. This approach not only maximizes the educational value of high-cost models but also contributes to overall cost-effectiveness in the implementation of robotic training programs.

Crucially, each step of the pathway should be anchored to clearly defined performance metrics and common critical errors specific to the target procedure, ensuring that all components coherently contribute to the achievement of proficiency by the trainee. Nevertheless, further evidence is needed to determine, more precisely, the number of repetitions and the amount of time required to progress from one stage to the next. Constructing the validity of this structured training model represents a key priority for future research.

The overall methodological quality of the included studies was acceptable, with all scoring in the moderate range on the MERSQI scale. However, the limited number of formal assessments, the heterogeneity of study designs, and the small sample sizes in some studies limit the generalizability of the findings. This introduces potential selection and publication bias, as smaller, single-institution studies may be more prone to confounding variables and less generalizable to broader surgical populations. Moreover, few studies addressed long-term retention of skills or performance in live clinical practice, which are essential endpoints in surgical education. Finally, the reliance on subjective assessments and the absence of blinded outcome evaluation in many studies further limit the reliability of their findings. To strengthen the evidence base, future research should prioritize multicenter RCTs with larger sample sizes and standardized outcome measures, ideally incorporating validated assessment tools and standardized metrics to reduce bias and improve reproducibility.

Given the growing adoption of robotic platforms in abdominal wall surgery, the field now faces an urgent need to move beyond fragmented and center-specific training approaches. It is time to establish a structured, internationally endorsed training pathway. PBP training has been shown to enhance the quality and safety of surgical education by emphasizing performance over repetition. Unlike traditional models, where procedural completion or time may be used as proxies for skill, PBP focuses on predefined, objective benchmarks that reflect expert-level execution. This includes not only the completion of procedural steps but the minimization of errors—both of which directly impact the quality of performance and patient safety [14, 15, 17].

In the context of robotic abdominal wall surgery, where technical demands are high and variability in access to the robotic platform exists, this approach offers clear advantages. By defining what constitutes both correct task execution and common performance errors, PBP allows for standardized, reproducible training across institutions. Crucially, trainees must demonstrate proficiency in simulation—using inanimate models, virtual reality platforms, or animal labs—before transitioning to live operative settings. This ensures that only individuals who have objectively met safety and performance standards proceed to patient care.

Moving forward, expert consensus—ideally established through Delphi methodology—should guide the definition of procedural steps and critical errors in robotic hernia surgery. These metrics would serve as the foundation for a structured, global training curriculum. Once validated, such a model could be adopted internationally, with societies like the European Hernia Society (EHS) playing a key role in certifying proficiency [40]. In this way, PBP has the potential to not only improve individual performance but also standardize training, reduce variability, and ultimately improve patient outcomes.

Robotic abdominal wall surgery requires structured training to ensure safe and effective practice. This review highlights the benefits of simulation-based and proficiency-based curricula, though evidence remains limited and heterogeneous. A standardized, stepwise training model—grounded in expert consensus and validated metrics—is essential to support widespread adoption. Preliminary data suggest that such training may improve outcomes while containing costs, particularly in complex cases. Future efforts should focus on curriculum validation, long-term outcomes, and formal certification to ensure global consistency in training standards.
