# Supplementary material for: Structured Training in Robotic Abdominal Wall Surgery: A Systematic Review of Educational Models, Methodologies, Existing Gaps and Unmet Needs
Source: J Abdom Wall Surg. 2025 Aug 18;4:15190. doi: 10.3389/jaws.2025.15190 (PMC12399436; doi:10.3389/jaws.2025.15190)
Supplement: Supplementary file 1 [file Supplementaryfile1.docx]

**PICO and complete search strategy**

**P (Population/Problem):** Surgeons or surgical trainees involved in abdominal wall surgery

**I (Intervention):** Training programs, simulation, or surgical education interventions

**C (Comparison) (if possible):** Traditional training or no training (optional depending on the research focus)

**O (Outcome):** Improved surgical skills, competence, safety, operative time, complication rate, etc.

**Total retrieved papers: 3038 (534 duplicates)**

**Pubmed (584)**

("Education"[Mesh] OR "Simulation Training"[Mesh] OR "training"[tiab] OR "Teaching"[Mesh] OR "Staff Development"[Mesh] OR "Preceptorship"[Mesh] OR "Curriculum"[Mesh] OR "curriculum"[tiab]) AND ("Robotic Surgical Procedures"[Mesh] OR "robotic" [tiab] OR "Robotics"[Mesh] OR "Endoscopy"[Mesh] OR endoscopic [tiab] OR "Natural Orifice Endoscopic Surgery"[Mesh] OR "Endoscopes"[Mesh] OR "Benchmarking"[Mesh]) AND ("Abdominal Wall Surgery"[tiab:~0] OR "Hernia Repair"[tiab:~0] OR "TAPP" [tiab] OR "Hernia" [tiab] OR "TEP" [tiab] OR "Transabdominal Preperitoneal" [tiab] OR "Totally Extraperitoneal" [tiab:~0] OR "Ventral Hernia"[tiab:~0])

**Embase (2421)**

('education'/exp OR 'simulation training'/exp OR training:ti,ab OR 'teaching'/exp OR 'staff development'/exp OR 'preceptorship'/exp OR 'curriculum'/exp OR curriculum:ti,ab)

AND

('robot assisted surgery'/exp OR robotic:ti,ab OR 'robotics'/exp OR 'endoscopy'/exp OR endoscopic:ti,ab OR 'natural orifice transluminal endoscopic surgery'/exp OR 'endoscope'/exp OR 'benchmarking'/exp)

AND

('abdominal wall surgery':ti,ab OR 'hernia repair':ti,ab OR tapp:ti,ab OR hernia:ti,ab OR tep:ti,ab OR 'transabdominal preperitoneal':ti,ab OR 'totally extraperitoneal':ti,ab OR 'ventral hernia':ti,ab)

**Cochrane (33)**

#1 (education:ti,ab,kw OR "simulation training":ti,ab,kw OR training:ti,ab,kw OR teaching:ti,ab,kw OR "staff development":ti,ab,kw OR preceptorship:ti,ab,kw OR curriculum:ti,ab,kw)

#2 AND ("robotic surgery":ti,ab,kw OR robotic:ti,ab,kw OR robotics:ti,ab,kw OR endoscopy:ti,ab,kw OR endoscopic:ti,ab,kw OR "natural orifice":ti,ab,kw OR endoscope:ti,ab,kw OR benchmarking:ti,ab,kw)

#3 AND ("abdominal wall surgery":ti,ab,kw OR "hernia repair":ti,ab,kw OR tapp:ti,ab,kw OR tep:ti,ab,kw OR hernia:ti,ab,kw OR "transabdominal preperitoneal":ti,ab,kw OR "totally extraperitoneal":ti,ab,kw OR "ventral hernia":ti,ab,kw)

#1 AND #2 AND #3

**Table 1s: ​​overview of the MERSQI and Kirkpatrick assessment frameworks**

| **Tool** | **Purpose** | **Domains / Levels** | **Scoring** |
| --- | --- | --- | --- |
| **MERSQI** | Assess methodological quality of educational studies | 6 domains: Study Design, Sampling, Type of Data, Validity, Data Analysis, Outcomes | Score range: 5–18 |
| **Kirkpatrick Model** | Evaluate training effectiveness | Level 1: Reaction  Level 2: Learning  Level 3: Behavior  Level 4: Results | Descriptive (not scored) |

**Table 2s: MERSQI scale**

| **Study** | **Study Design (0–3)** | **Sampling (0–3)** | **Type of Data (0–3)** | **Validity of Evaluation Instrument (0–3)** | **Data Analysis (0–3)** | **Outcomes (0–3)** | **Total Score (0–18)** |
| --- | --- | --- | --- | --- | --- | --- | --- |
| **Hays et al. (2024)** | **2** | **2** | **2** | **2** | **2** | **2** | **12** |
| **Vierstraete et al. (2024)** | **1** | **1** | **1** | **1** | **1** | **1** | **6** |
| **Gonçalves et al. (2023)** | **2** | **2** | **2** | **1** | **2** | **2** | **11** |
| **Ollapallil Jacob et al. (2023)** | **1** | **1** | **1** | **0** | **1** | **1** | **5** |
| **Ebeling et al. (2020)** | **2** | **1** | **2** | **1** | **1** | **2** | **9** |
| **Tam et al. (2019)** | **3** | **2** | **3** | **2** | **2** | **3** | **15** |
| **Moustafa et al. (2018)** | **1** | **2** | **2** | **1** | **1** | **2** | **9** |
| **Madureira et al. (2017)** | **2** | **2** | **2** | **1** | **2** | **2** | **11** |
